# Quality of Life and Metabolomics Analysis in Response to Meal Kit Intervention During Perioperative Chemotherapy in Patients with Early-Stage Breast Cancer: Protocol for a Single-Center Phase Ⅱ Randomized Crossover Trial

**DOI:** 10.2196/72715

**Published:** 2025-12-04

**Authors:** Atsushi Fushimi, Eriko Taguchi, Makiko Kamio, Takashi Kazama, Azusa Fuke, Hiroko Nogi

**Affiliations:** 1 Department of Breast and Endocrine Surgery, The Jikei University School of Medicine, 3-25-8 Nishi-Shinbashi, Minato-ku, Tokyo, 105-8461, Japan, 81 3-3433-1111 ext 3401

**Keywords:** nutritional intervention, perioperative treatment, patient-reported outcomes, metabolic profiling, supportive care

## Abstract

**Background:**

Patients with breast cancer undergoing chemotherapy experience significant adverse effects, including fatigue, nausea, and taste alterations, leading to malnutrition in 40% of patients. Traditional nutritional counseling has shown limited effectiveness in addressing these challenges during treatment.

**Objective:**

This study aimed to evaluate the impact of a structured meal kit intervention on quality of life and metabolomic profiles in patients with breast cancer during perioperative anthracycline and taxane-based chemotherapy.

**Methods:**

This single-center phase 2 randomized crossover trial will enroll 20 patients with breast cancer scheduled for perioperative chemotherapy at Jikei University Hospital between October 2024 and October 2025. Patients will be randomized 1:1 to receive a meal kit intervention either during the first or second 3-month period of chemotherapy. The intervention consists of weekly deliveries of preportioned ingredients with dietitian-designed recipes (one 2-serving meal kit during chemotherapy weeks, two during nonchemotherapy weeks). The primary endpoint is the change in the EORTC QLQ-C30 (European Organisation for Research and Treatment of Cancer Quality of Life Questionnaire Core 30) “appetite loss” domain score. Secondary endpoints include changes in total EORTC QLQ-C30 score, plasma metabolome profiles, body composition, nutritional status, and chemotherapy completion rate. Data will be collected at baseline, crossover (12 wk), and study completion (24 wk).

**Results:**

The study was funded in March 2024. Data collection began on March 26, 2025, and as of manuscript submission, 11 participants have been recruited out of the planned 20 participants. Data analysis has not yet commenced, and results are expected to be published in Spring 2026.

**Conclusions:**

This trial will provide evidence for the effectiveness of a meal kit intervention in supporting patients with breast cancer during chemotherapy. The findings may help establish evidence-based standards for nutritional support in oncology care.

## Introduction

Breast cancer is the leading cancer among Japanese women, with approximately 95,000 new cases diagnosed annually [1]. About 30% of early patients with breast cancer receive chemotherapy as part of their treatment [1]. While chemotherapy improves survival outcomes, treatment-related side effects significantly impact patients’ quality of life (QOL) and nutritional status. Studies have consistently shown that malnutrition is prevalent among patients with cancer undergoing chemotherapy, affecting 40% of patients [2,3]. Malnutrition is associated with poorer QOL outcomes, including reduced physical, emotional, and cognitive functioning. Common side effects impacting nutritional status include fatigue, nausea, vomiting, changes in taste and smell, and gastrointestinal disturbances [4,5]. These side effects can lead to inadequate nutrient intake and further deterioration of nutritional status [6].

Poor nutritional status during chemotherapy is associated with decreased treatment adherence, increased adverse events, and higher health care consumption [7]. A systematic review of 71 studies found a significant association between poor nutritional status and increased mortality (hazard ratio 1.87, 95% CI 1.62‐2.17), with malnourished patients being less likely to complete oncologic treatment according to plan [7]. Cross-sectional studies have shown that malnutrition during treatment correlates with lower QOL scores across all functional domains [3]. Despite this evidence, structured nutritional interventions during chemotherapy remain limited, with most support being generic dietary advice. Recently, Tang et al [8] reported a protocol for a multidisciplinary early intervention during chemotherapy in patients with breast cancer, highlighting the importance of structured support for dietary behavior and quality of life management from the early phase of treatment.

Meal kit interventions represent an innovative approach to addressing these challenges. Unlike traditional nutritional counseling, meal kits provide preportioned ingredients and recipes tailored to patients’ needs, potentially improving dietary adherence and reducing the burden of meal preparation during treatment [6]. Recent studies have demonstrated promising results with nutritional interventions during chemotherapy, showing improvements in dietary intake, quality of life, and symptom management [9]. A randomized controlled trial of plant-based, high-protein meal kits showed significant improvements in fatigue scores and body composition measurements compared to standard nutritional counseling [10], while another study of Mediterranean Diet meal delivery found that home delivery services combined with nutritional education can facilitate better dietary adherence, though chemotherapy-induced side effects remain a significant barrier [11]. However, more comprehensive randomized trials are needed to fully evaluate the effectiveness of meal kit interventions specifically for patients with breast cancer during chemotherapy.

The present trial aims to fill these research gaps through three unique aspects. First, it used a randomized crossover design to evaluate the meal kit intervention during both anthracycline and taxane-based chemotherapy phases. Second, it combines patient-reported QOL outcomes with comprehensive metabolomics analysis to understand the biological impact of nutritional support. Third, it assesses the feasibility and effectiveness of a structured meal kit program that could be implemented in routine clinical practice.

This study will provide critical evidence for developing evidence-based nutritional support strategies during breast cancer chemotherapy. The findings may help establish standards for personalized dietary interventions and inform future large-scale implementation studies.

## Methods

### Ethical Considerations

The study protocol has been approved by the Institutional Review Board of Jikei University School of Medicine (approval number: 36-088[12191]). Written informed consent will be obtained from all participants. Results will be published in peer-reviewed journals and presented at scientific conferences. Patients were not involved in the development of the research question or study design. Study results will be disseminated to participants through a summary report.

### Study Design and Setting

This is a single-center phase 2 randomized crossover trial at the Department of Breast and Endocrine Surgery, Jikei University Hospital, Tokyo, Japan. The study period is from October 2024 to December 2026, with patient registration from October 2024 to December 2025. This crossover design was selected based on established approaches for nutritional interventions in cancer patients, which maximize statistical efficiency by having each patient serve as their own control [12].

### Participants

Eligible participants are women aged 20 years or older with histologically confirmed stage 0–III breast cancer who are scheduled to receive perioperative chemotherapy consisting of anthracycline- and taxane-based regimens. Participants must have an Eastern Cooperative Oncology Group (ECOG) Performance Status of 0‐1 and be able to provide written informed consent. Patients with severe food allergies, serious comorbidities, or conditions preventing safe meal preparation will be excluded. Additional exclusion criteria include pregnancy or lactation and inability to understand or provide informed consent. These eligibility criteria are consistent with prior nutritional intervention and supportive care trials conducted in breast cancer patients undergoing chemotherapy [13-16].

### Intervention

The meal kit intervention consists of weekly deliveries of nutritionally balanced, preportioned meal kits developed by registered dietitians in collaboration with oncology clinicians. These kits are part of the “Healthcare Oisix” program, a food support service specifically designed for cancer patients undergoing chemotherapy.

Each meal kit includes precut ingredients, seasonings, and recipe cards, and is designed to align with core principles of evidence-based nutritional support during chemotherapy. While current international guidelines do not recommend fixed per-meal nutritional targets, our kits are structured to reflect best practices derived from clinical trials and consensus recommendations, emphasizing key elements such as:

- High-quality protein content (~25‐30 g/meal) to approximate 1.2‐1.5 g/kg/day, supporting lean body mass preservation and mitigating cachexia during chemotherapy [13-15]
- Adequate energy density (400‐500 kcal/meal) to help maintain energy balance, especially in patients experiencing appetite loss [17,18]
- Digestive tolerability, through soft-textured, low-fiber, easily digestible components (eg, tofu, simmered fish, soft vegetables), reduces mucosal irritation and improves intake in patients with gastrointestinal symptoms [19,20]
- Anti-inflammatory potential, incorporating Mediterranean-style elements such as olive oil, fish, and beans, which may modulate inflammatory markers and support overall resilience [21,22]
- Flavor optimization, through seasoning variety and familiar ingredients, to address taste alterations and promote appetite and food enjoyment during treatment [23-25]

In addition to the main dishes, supplementary items such as nutritional jelly, yogurt, soy milk, and protein-rich snacks (eg, cheese, tofu, chicken flakes) are included weekly to improve dietary adherence and intake consistency.

All meals are designed to require minimal preparation and cognitive demand, accommodating patients experiencing fatigue or chemotherapy-induced cognitive changes. Patients are instructed to log all consumed items weekly using a structured food diary to track adherence and enable dietary intake assessment.

During chemotherapy weeks and the following week, patients receive one 2-serving meal kit. During the subsequent week, they receive two 2-serving meal kits. Over 3 months, each patient receives 16 meal kits total.

### Randomization and Blinding

Block randomization (block size=2) will be performed using a central registration system. The allocation sequence will be computer-generated by an independent statistician and concealed until interventions are assigned. Due to the nature of the intervention, blinding is not feasible. However, to minimize potential bias, several mitigation strategies will be implemented. Objective measures such as metabolomic profiles, anthropometric data, and laboratory values will serve as secondary validation of patient-reported outcomes. In addition, validated and structured questionnaires will be used to reduce reporting variability and enhance the reliability of self-reported data.

In this hospital, nearly all patients undergoing perioperative chemotherapy for breast cancer receive a standard regimen consisting of anthracycline-based treatment followed by taxane-based treatment. This reflects current clinical practice and minimizes heterogeneity in chemotherapy exposure. Therefore, the diversity of chemotherapy regimens is unlikely to introduce significant confounding. Nevertheless, randomization will be stratified by planned chemotherapy regimen (anthracycline-taxane sequence vs anthracycline-taxane with targeted therapy), and regimen type will be included as a covariate in the statistical models to account for any residual variability.

### Outcomes

The primary outcome of this study is the change in the EORTC QLQ-C30 (European Organisation for Research and Treatment of Cancer Quality of Life Questionnaire Core 30) “appetite loss” domain score between the meal-kit intervention and standard care periods. Key secondary outcomes include changes in the total EORTC QLQ-C30 global health and functional scores, body weight, body mass index, body composition, and chemotherapy completion rate, including relative dose intensity. Exploratory outcomes comprise changes in plasma metabolomic profiles, nutritional status evaluated by the Subjective Global Assessment, and laboratory parameters such as serum albumin, hemoglobin, zinc, and vitamin D levels. Additional exploratory analyses will assess adverse event rates, dietary intake, and nutrient composition, as well as patient-reported measures of meal satisfaction and adherence. Exploratory outcomes will be analyzed descriptively, with multiple comparisons adjusted using the Benjamini–Hochberg method.

### Sample Size

As an exploratory phase 2 trial designed to generate preliminary data for future larger-scale randomized controlled trials, we plan to enroll 20 patients (10 per sequence group). This sample size was determined based on feasibility constraints for this single-center pilot study, similar to other exploratory nutritional intervention studies in oncology settings [16]. The crossover design maximizes statistical efficiency by having each patient serve as their own control, reducing interindividual variability in dietary habits, metabolic profiles, and treatment tolerance. These preliminary data will inform power calculations for future confirmatory phase 3 trials.

### Chemotherapy Regimens

#### Anthracycline-based chemotherapy:

- Doxorubicin 60 mg/m2 Day1+Cyclophosphamide 600 mg/m2 Day1 q3w, 4 cycles, or
- Doxorubicin 60 mg/m2 Day1+Cyclophosphamide 600 mg/m2 Day1+Pembrolizumab 200 mg Day1 q3w, 4 cycles

#### Taxane-based chemotherapy:

Taxane-based chemotherapy:

- Paclitaxel 80 mg/m2 Day1,8,15 q3w, 4 cycles
- Docetaxel 75 mg/m2 Day1 q3w, 4 cycles
- With or without Trastuzumab, Pertuzumab, or Pertuzumab-Trastuzumab-Hyaluronidase-zzxf, or
- Carboplatin AUC1.5 Day1,8,15+Paclitaxel 80 mg/m2 Day1,8,15+Pembrolizumab 200 mg Day1 q3w, 4 cycles

### Study Period

The registration period will be from October 2024 to December 2025; the study period will run from October 2024 to December 2026; and the follow-up period will continue until May 2026.

### Data Sharing Statement

Deidentified participant data will be available upon reasonable request after study completion. Requests should be directed to the corresponding author. Data will be available for 5 years after publication.

### Data Collection and Management

Clinical data will be collected from electronic medical records. Blood samples will be stored at −80°C until metabolomic analysis. QOL assessments will be conducted every 3 weeks during hospital visits. All data will be managed using a secure database with coded identifiers to protect patient privacy.

### Dietary Adherence Assessment

Dietary adherence will be monitored through food consumption logs where participants record what they consumed from each meal kit. We will also conduct dietary habit change questionnaires to assess how the meal kit intervention influences participants’ overall eating patterns compared to baseline. Meal satisfaction surveys will evaluate ease of preparation and palatability as indicators of adherence likelihood.

Adherence will be defined as consumption of meal kit components during at least 80% of delivered weeks. This approach allows evaluation of both direct meal kit consumption and broader dietary behavioral changes resulting from the intervention.

### Carryover Effects Assessment

While a traditional washout period is not feasible due to the continuous nature of chemotherapy treatment, several strategies will be employed to assess and mitigate carryover effects.

1. Statistical analysis will include testing for period effects and treatment-by-period interactions using linear mixed-effects models
2. Sensitivity analyses will be conducted, excluding the first week of each period, to minimize immediate carryover effects
3. Baseline measurements will be monitored at each crossover point to detect persistent effects from the previous period
4. The analysis will specifically examine whether treatment effects differ between the first and second treatment periods

### Metabolomics Analysis

Plasma metabolomic analysis will be performed using gas chromatography-mass spectrometry (GC-MS), a validated platform for detecting metabolic alterations in breast cancer patients undergoing chemotherapy or nutritional intervention. GC-MS has identified changes in amino acid (eg, glutamate, lysine, threonine), lipid, and energy metabolism (eg, lactate, hydroxybutyrate), reflecting tumor progression and treatment response [26,27]. Compared to other platforms, GC-MS offers superior resolution for volatile and small metabolites, complementing broader lipid and polar compound coverage by liquid chromatography-mass spectrometry and nuclear magnetic resonance [28].

### Statistical Analysis

Primary analysis will use linear mixed-effects models for repeated measures, with treatment, period, sequence, and treatment-by-period interaction as fixed effects and subject as a random effect. The analysis will test for treatment effects comparing meal kit versus standard care, period effects between first and second treatment periods, and sequence effects with potential carryover effects assessed through treatment-by-period interactions. Missing data will be handled using multiple imputation. If significant carryover effects are detected, sensitivity analyses will be conducted using first-period data only. Metabolomic data will be analyzed using principal component analysis and partial least squares discriminant analysis. The Benjamini-Hochberg method will be used to control for multiple comparisons. Sensitivity analyses will include per-protocol analysis excluding participants with less than 80% adherence, analysis excluding the first week of each treatment period, first-period only analysis if carryover effects are detected, and subgroup analyses by chemotherapy regimen type if sample size permits.

This protocol was developed in accordance with the SPIRIT (Standard Protocol Items: Recommendations for Interventional Trials) 2025 statement for randomized trial protocols. The completed SPIRIT 2025 checklist is available as Checklist 1.

## Results

This study was funded in March 2024 by Oisix ra daichi Co., Ltd. Enrollment began on March 26, 2025, following approval by the Institutional Review Board of The Jikei University School of Medicine. As of manuscript submission (July 31, 2025), 11 participants have been recruited and randomized out of the planned 20 participants. Data collection is ongoing with an expected completion date of March 2026. Data analysis will commence upon completion of data collection, with results expected to be published in Spring 2026.

## Discussion

This trial represents a novel approach to assessing the impact of a meal kit intervention on the nutritional status and quality of life of breast cancer patients undergoing chemotherapy. Chemotherapy, while essential for improving survival outcomes, is often accompanied by adverse effects such as fatigue, nausea, and alterations in taste that contribute to malnutrition and diminished quality of life. Traditional nutritional counseling may be insufficient to address these challenges, particularly when patients struggle with the practical aspects of meal preparation during treatment. In contrast, our intervention, which provides preportioned ingredients and standardized recipes designed by registered dietitians, is designed to alleviate these burdens and promote better dietary adherence.

The randomized crossover design of this study allows each patient to serve as their own control, thereby reducing the confounding effects of individual variability in dietary habits, metabolic profiles, and treatment tolerance, similar to the approach used in other nutritional intervention studies [29]. This design allows us to compare the effects of meal kit intervention during both anthracycline and taxane phases within the same patient, which is particularly advantageous given the exploratory nature of this phase 2 trial and the relatively small sample size of 20 patients. By aligning the intervention with these distinct chemotherapy phases, we are able to evaluate the effects of the meal kit on appetite loss and other QOL domains, as previous research has shown that different chemotherapy regimens are associated with distinct side effect profiles affecting nutritional status [30].

A significant strength of this study lies in its comprehensive assessment of both patient-reported outcomes and objective biological measures. The integration of quality of life assessments using the EORTC QLQ-C30, which has been validated as an effective tool for measuring QOL in cancer patients [31], with metabolomic analyses via gas chromatography-mass spectrometry provides a multidimensional perspective on the intervention’s impact. This integrated approach, combining patient-reported outcomes with metabolomic data, has shown promise in previous research for understanding treatment effects [32], and may yield valuable insights into the biological mechanisms underlying the relationship between nutritional intake and symptom management during chemotherapy, similar to previous metabolomic studies in breast cancer patients [33].

Despite these strengths, several limitations must be acknowledged. As an exploratory phase 2 trial conducted at a single center with 20 participants, the statistical power to detect subtle effects is limited, and the generalizability of the findings may be constrained. While the inability to blind participants and treatment providers to the intervention introduces the potential for reporting bias in the quality of life measures, we incorporate objective measures such as metabolomic analyses and standardized assessments to strengthen our findings. Furthermore, the six-month follow-up period, while sufficient for evaluating acute effects, does not allow for the assessment of long-term outcomes such as treatment adherence, survival rates, or sustained quality of life improvements.

Future research should build upon this phase 2 trial through larger, multicenter randomized controlled trials that can definitively establish the role of meal kit interventions in oncology care. Studies with extended follow-up periods would allow evaluation of not only immediate effects on quality of life and metabolic profiles, but also potential impacts on treatment adherence and long-term survival outcomes. While our statistical models will account for chemotherapy regimen type, future research could specifically explore whether the timing of meal kit intervention (during anthracycline vs taxane phase) differentially affects outcomes, as the impact of nutritional support may vary across different chemotherapy regimens. Additionally, investigating the integration of meal kits with other supportive care strategies could reveal synergistic benefits for symptom management and patient well-being. Such comprehensive research would help establish evidence-based standards for nutritional support during chemotherapy and potentially transform the approach to supportive care in oncology.

In conclusion, this study offers a systematic approach to evaluating a standardized meal kit intervention in breast cancer patients undergoing chemotherapy. By addressing both the practical and biological challenges of maintaining adequate nutrition during treatment through a structured program of preportioned ingredients and registered dietitian-designed recipes, our findings have the potential to inform the development of evidence-based nutritional support strategies that enhance quality of life and optimize clinical outcomes in oncology.

## Supplementary material

10.2196/72715 Checklist 1 SPIRIT 2025 editable checklist.

## Abbreviations

ECOG: Eastern Cooperative Oncology Group
EORTC-QLQ-C30: European Organisation for Research and Treatment of Cancer Quality of Life Questionnaire Core 30
GC-MS: gas chromatography-mass spectrometry
QOL: quality of life
SPIRIT: Standard Protocol Items: Recommendations for Interventional Trials
